# Eysenck's Theory of Personality and the Role of Background Music in Cognitive Task Performance: A Mini-Review of Conflicting Findings and a New Perspective

**DOI:** 10.3389/fpsyg.2017.01991

**Published:** 2017-11-14

**Authors:** Mats B. Küssner

**Affiliations:** Institut für Musikwissenschaft und Medienwissenschaft, Humboldt-Universität zu Berlin, Berlin, Germany

**Keywords:** Eysenck's theory of personality, background music, extraversion, cognitive task performance, EEG, alpha/beta band power, arousal, focused listening

## Abstract

The question of whether background music is able to enhance cognitive task performance is of interest to scholars, educators, and stakeholders in business alike. Studies have shown that background music can have beneficial, detrimental or no effects on cognitive task performance. Extraversion—and its postulated underlying cause, cortical arousal—is regarded as an important factor influencing the outcome of such studies. According to Eysenck's theory of personality, extraverts' cortical arousal at rest is lower compared to that of introverts. Scholars have thus hypothesized that extraverts should benefit from background music in cognitive tasks, whereas introverts' performance should decline with music in the background. Reviewing studies that have considered extraversion as a mediator of the effect of background music on cognitive task performance, it is demonstrated that there is as much evidence in favor as there is against Eysenck's theory of personality. Further, revisiting Eysenck's concept of cortical arousal—which has traditionally been assessed by activity in the EEG alpha band—and reviewing literature on the link between extraversion and cortical arousal, it is revealed that there is conflicting evidence. Due to Eysenck's focus on alpha power, scholars have largely neglected higher frequency bands in the EEG signal as indicators of cortical arousal. Based on recent findings, it is suggested that beta power might not only be an indicator of alertness and attention but also a predictor of cognitive task performance. In conclusion, it is proposed that focused music listening *prior* to cognitive tasks might be a more efficient way to boost performance than listening to background music during cognitive tasks.

## The effects of background music on cognitive task performance

There has been a surge in commercial applications promising to improve their users' concentration and focus by playing specifically designed music in the background. The basic idea is simple: playing background music activates your brain and leads to better performance in cognitive tasks. However, this idea comes with several issues. For example, even if music is specifically designed to set free cognitive resources it is implausible that every person benefits in the same manner in a cognitive task. A piece of music that has beneficial effects on cognitive task performance for one individual may well have no effect, or even detrimental effects, for another. Is searching for background music that enhances cognitive task performance therefore chasing a red herring? Not necessarily, if—as a first step—we are able to identify and better understand the neural underpinnings that enhance cognitive task performance in general. As a second step, we may begin to ask what are the characteristics of the music needed to change an individual's neural activation in a specific way. Since inter-individual differences play an important role in this endeavor, the evidence in favor of, and against, Eysenck's theory of personality will be reviewed before a new perspective is provided.

Although the effects of background music on cognitive task performance have been studied by psychologists and educators for more than seventy years (Fendrick, 1937), no clear pattern of results has emerged thus far. On the one hand, background music, in comparison with silence, has been found to be beneficial for reading comprehension (Kiger, 1989), foreign vocabulary learning (de Groot, 2006; Kang and Williamson, 2014), spatial and linguistic processing (Angel et al., 2010), IQ tests (Cockerton et al., 1997), spatial and numerical reasoning (Miller and Schyb, 1989), visual search tasks (Crust et al., 2004), and students' achievement in a psychology class (Schreiber, 1988). On the other hand, background music, compared to silence, has been found to impair cognitive performance, showing detrimental effects for reading comprehension (Fendrick, 1937; Henderson et al., 1945; Etaugh and Ptasnik, 1982; Furnham and Bradley, 1997; Avila et al., 2012; Thompson et al., 2012), verbal memory (Iwanaga and Ito, 2002; Woo and Kanachi, 2005; Cassidy and MacDonald, 2007), visual memory (Furnham and Bradley, 1997), serial recall of digits (Nittono, 1997; Alley and Greene, 2008), Stroop tasks (Parente, 1976; Cassidy and MacDonald, 2007), writing fluency (Ransdell and Gilroy, 2001), and logical reasoning and associative learning (Crawford and Strapp, 1994). Yet other investigations revealed that background music had no significant impact on cognitive task performance whatsoever (Henderson et al., 1945; Freeburne and Fleischer, 1952; Furnham and Allass, 1999; Pool et al., 2003; Alley and Greene, 2008; Schlittmeier and Hellbrück, 2009; Thompson et al., 2012). A recent meta-analysis on the effects of background music on adults' cognitive, affective and behavioral responses seems to support the trend toward an overall null effect (Kämpfe et al., 2011).

Without theory-driven research focused on inter-individual differences, these contradictory findings are not surprising. To address this issue, many scholars have used Eysenck's theory of personality (Eysenck, 1967) as a theoretical framework for their studies. While there are several inter-individual differences that influence the effects of background music on cognitive task performance—ranging from personality traits to musical taste and age—one inter-individual difference that has been studied extensively is extraversion.

According to a particular aspect of Eysenck's theory of personality, extraversion can be described and explained by the underlying cortical arousal. Extraverts are reported to have a lower level of cortical arousal compared to introverts. Eysenck's theory therefore predicts that introverts require little or no external stimulation to reach an optimal level of cognitive performance, whereas extraverts require comparatively more external stimulation. External stimulation exceeding the optimal threshold should lead to a decline in cognitive performance, following the Yerkes-Dodson Law (Yerkes and Dodson, 1908). Thus, presented with moderate to high levels of external stimulation should lead to a decline in introverts', but not extraverts', cognitive performance.

Using background music as a source of external stimulation—which has been shown to increase arousal in participants in several studies (Thompson et al., 2001; Jones et al., 2006; Schellenberg et al., 2007)—researchers have empirically tested Eysenck's theory by investigating intro- and extraverts' performance in different cognitive tasks.

## Introverts' and extraverts' performance in cognitive tasks with background music and silence

There is a substantial amount of evidence in favor of Eysenck's theory of personality, as the following studies show. Reporting a clear cross-over interaction between extraversion and background condition (either silence, simple music or complex music), Furnham and Allass (1999) showed that introverts' performance in two memory tests—immediate and delayed recall of visual objects—was best during silence and poorest with complex music, whereas extraverts performed best with complex music and poorest during silence. However, most evidence supporting Eysenck's theory of personality reveals music's detrimental effect on introverts' performance rather than music's beneficial effect on extraverts' performance in comparison with silence. For instance, testing introverts and extraverts during silence or with pop music in the background, Furnham and Bradley (1997) found that introverts who carried out a memory test in silence performed better than introverts presented with pop music. The same authors also showed that introverts completing a reading comprehension task performed poorer in the presence of music compared to silence, whereas extraverts showed no difference. Other studies have provided similar findings for reading comprehension tasks: Daoussis and McKelvie (1986) reported that introverts showed a poorer performance with rock'n'roll music in the background compared to silence, whereas extraverts did not differ in these two conditions. Furnham and Strbac (2002) showed that introverts performed poorer with music or office noise in the background compared to silence, whereas no difference between these three conditions was found in extraverts. Other cognitive tasks have produced comparable results. Introverts showed a linear decline in performance from silence to simple, and then complex, music in a spatial reasoning task (Furnham and Allass, 1999), and Cassidy and MacDonald (2007) revealed that, compared to silence, the presence of highly arousing music with negative affect—as well as the presence of background noise—led to a poorer performance of introverts compared to extraverts in a Stroop task. Another study (Dobbs et al., 2011) showed that extraversion was a significant predictor of performance in an abstract reasoning task and in a test of general cognitive ability when music or noise was present in the background. The more introvert a participant was the poorer their performance under these conditions—especially during noise—whereas task completion in silence revealed no, or only very weak, differential effects. Further indirect evidence was provided by Crawford and Strapp (1994) who tested a sample of students reporting usually studying either with or without background music. Those studying without background music showed a linear decline in performance in an associative memory task from silence to instrumental, and then vocal, music, whereas those studying with background music showed no clear pattern. In accordance with Eysenck's theory of personality, the latter group scored significantly higher on an extraversion scale than those studying without background music.

Even though these studies form a substantial body of evidence in favor of Eysenck's theory of personality, there are also several studies that have failed to support his theory. Testing intro- and extraverts, neither Furnham et al. (1999) nor Avila et al. (2012) found a significant interaction between extraversion and background condition—either vocal music, instrumental music or silence—in any of the following tests: reading comprehension tasks, logical reasoning, a coding task, a numerical test or a diagrammatic test (see also Furnham and Allass, 1999; Kou et al., 2017). Nor did Chamorro-Premuzic et al. (2009) find such interaction effect in logical reasoning or verbal tasks. Absence of the interaction between extraversion and background condition was further documented in arithmetic tasks and prose recall (Furnham and Strbac, 2002), and the hypothesized interaction was also absent in various memory tasks: immediate, delayed and free recall of verbal items (Cassidy and MacDonald, 2007), as well as immediate recall of visual objects (Furnham and Bradley, 1997).

Although taking into account inter-individual differences is vital when studying the effects of background music on cognitive task performance, the conflicting findings seem to suggest that extraversion, as measured with standard questionnaires alone, does not lead to conclusive results (see overview in Table 1). In an attempt to start disentangling these mixed findings, researchers have considered a more objective way of assessing inter-individual differences, i.e., investigating what Eysenck regarded as the underlying cause of differences in extraversion: cortical arousal (for a review see Matthews and Gilliland, 1999).

**Table 1:** Studies testing intro- and extraverts' performance in cognitive tasks under various background conditions.

**Study**	**N**	**Experimental Conditions**	**Task**	**Support for Eysenck's theory of personality**
Avila et al., 2012	58	Vocal music / instrumental music / silence	Verbal test	No
			Numerical test	No
			Logic test	No
Cassidy and MacDonald, 2007	40	High arousal, negative music / low arousal, positive music / everyday noise/silence	Immediate recall	No
			Free recall	No
			Delayed recall	No
			Stroop task	Yes
Chamorro-Premuzic et al., 2009	77	Speech / noise / music / silence	Logical reasoning test	No
			Sentence-completion test	No
			Alternate-uses test	Yes
Crawford and Strapp, 1994	61	Vocal music / instrumental music / silence	Scanning speed	No
			Logical reasoning	No
			Associative memory	Yes (indirectly)
Daoussis and McKelvie, 1986	48	Rock'n'roll music / silence	Reading recall test	Yes
Dobbs et al., 2011	118	UK garage music / background noise / silence	Abstract (perceptual) reasoning	Yes
			General cognitive ability	Yes
			Verbal reasoning test	Yes
Furnham and Allass, 1999	48	Complex musical distraction / simple musical distraction / silence	Reading comprehension test	No
			Observation test	Yes
			Immediate recall	Yes
			Delayed recall	Yes
Furnham and Bradley, 1997	20	Pop music / silence	Reading comprehension test	Yes
			Immediate recall	No
			Delayed recall	Yes
Furnham and Strbac, 2002	76	Garage music / office noice / silence	Reading comprehension task	Yes
			Prose recall task	No
			Mental arithmetic task	No
Furnham et al., 1999	144	Vocal music / instrumental music / silence	Reading comprehension task	No
			Logic problem task	No
			Coding task	No
Kou et al., 2017	92	Chinese pop songs / office noise / silence	Abstract (perceptual) reasoning	No
			Reading comprehension test	No
			Arithmetic test	No
Küssner et al., 2016	69	Baroque music / silence	Foreign vocabulary learning	No

## Extraversion and cortical arousal in the EEG alpha and beta bands

Eysenck himself (Hagemann et al., 1999) suggested that differences in extraversion are reflected in the basal level of cortical arousal, hypothesizing that extraverts possess a lower basal level compared to introverts. Traditionally, cortical arousal is measured as alpha power in the electroencephalogram. Researchers have long held the view that low alpha power (8–13 Hz) is associated with high mental activity (Ray and Cole, 1985; Schmidtke and Heller, 2004). In other words, more alpha power is an indicator of an idle neural state. Although Ray and Cole (1985) have argued that this arousal model simplifies the actual mechanisms by providing evidence that alpha power is related to attentional processes, whereas beta power (14–35 Hz)—normally associated with wakefulness and alertness—reflects emotional or cognitive processes, alpha power is still often used as a measure of cortical arousal, possibly because Eysenck himself (1994, p. 167, as cited in Matthews and Gilliland, 1999) regarded EEG, and particularly alpha power, as the “standard measure of cortical arousal.”

There is empirical support for Eysenck's claims. For instance, in a study measuring the same participants' basal cortical arousal three times over the course of several weeks to ensure variance introduced by external factors such as time of day or emotional events is minimized, Hagemann and colleagues (Hagemann et al., 2009) revealed that extraverts show more alpha power (i.e., less cortical arousal) than introverts. Similarly, Gale et al. (1969) reported more activity in the lower alpha range (7.5–10.5 Hz) in extraverts compared to introverts during a baseline measure of cortical arousal with eyes closed, as well as more alpha power using a gross filter (8–13 Hz) during moderate levels of external visual stimulation. Asking participants to empathize with positive and negative facial expressions while recording EEG data, Gale et al. (2001) showed again more alpha power in the lower (8–10 Hz) but not the higher alpha (10–12 Hz) band in extraverts compared to introverts. More support for Eysenck's theory of personality comes from Smith et al. (1995) who reported that introverts show less activity in the alpha band (i.e., more cortical arousal) than extraverts during presentation of positive, negative or neutral non-verbal auditory stimuli.

On the other hand, only weak evidence in favor of Eysenck's theory was provided by Beauducel et al. (2006) who found no significant effect in extraverts during a 40-min monotonous vigilance task. Furthermore, using baseline measures of cortical arousal, neither Hagemann et al. (1999) nor Schmidtke and Heller (2004) were able to find a relationship between alpha power and extraversion. Furthermore, Matthews and Amelang (1993) found no association between alpha power and extraversion, neither in any of three experimental conditions separately—silence with eyes closed, visual fixation and mental arithmetic with eyes closed—nor averaged across them.

Taken together, these findings suggest that alpha power might not be the most appropriate frequency range as an indicator of cortical arousal, especially during baseline levels of arousal. A possibly better alternative but less understood measure of cortical arousal is beta power as suggested by Ray and Cole (1985). Several studies have used beta power as an indicator of cortical arousal or alertness (Gale et al., 1969; Matthews and Amelang, 1993; Cardenas et al., 1997; Rangaswamy et al., 2002; Gram et al., 2005). Regarding cortical arousal and extraversion three studies are relevant here. Gale et al. (1969) reported more beta power in extraverts than introverts during a baseline measure of cortical activity with eyes closed. Similarly, Matthews and Amelang (1993) found a positive correlation between extraversion and beta power during a condition of moderate external stimulation. Importantly, these two findings are *opposite* to what one might expect based on Eysenck's theory of personality. Since high beta is supposed to be related to high cortical arousal, one would expect extraverts to show *less* beta power than introverts. Matthews and Amelang suggest that this assumption—high beta associated with high cortical arousal—could be wrong and speculate that high cortical arousal might as well be indicated by low beta power. However, there is at least one study that shows the predicted relationship. Gram et al. (2005) tested intro- and extraverts during a 2-min baseline condition with eyes open. In line with Eysenck's theory of personality the authors showed that extraverts exhibit more alpha power and less beta power than introverts. The distinction between the two groups was clearly present in higher beta bandwidths (26–39 Hz), but less clear in low beta (13–25 Hz), suggesting that finer beta filters might be needed to find the hypothesized relationship between beta activity and extraversion.

To sum up, it should have become obvious that no clear link between extraversion and cortical arousal currently exists. Although alpha power has traditionally been used as an indicator of cortical arousal to distinguish between intro- and extraverts, this might not be the most suitable neural correlate of cortical arousal. Given the contradictory literature on the effects of background music on cognitive task performance, the relationship between extraversion, alpha power and task performance is obscure. What might elucidate this situation is beta power as an indicator of cortical arousal, even though the role of beta power for cognitive task performance, let alone its relationship with background music, is still poorly understood.

## A new perspective: music, beta power and cognitive task performance

There is evidence that beta power increases during cognitive tasks, e.g., in a sustained attention test (Molteni et al., 2007) or during reading and subtraction tasks (Fitzgibbon et al., 2004). What is more, a recent study provided evidence that more beta power is associated with enhanced cognitive performance. Küssner et al. (2016) showed that beta power predicts the number of correctly recalled words in a foreign-vocabulary learning task. EEG was measured in silence before the learning task, thus hinting at the importance of the level of cortical arousal *prior* to a learning task. The authors also used background music to induce cortical arousal in extra- and introverts but found no evidence in favor of Eysenck's theory of personality.

The effect of beta power on cognitive task performance opens up new questions. First, we need a better understanding of the role of beta power in cognitive task performance. Due to Eysenck's focus on alpha power, many scholars have used alpha rather than beta power to assess cortical arousal, neglecting higher frequency bands in the EEG signal. To investigate empirically whether beta power predicts performance one could measure beta power as a predictor in a regression model with cognitive task performance as outcome variable. Secondly, if beta power turns out to be a significant predictor of performance—perhaps even in different types of cognitive tasks—a logical follow-up question is: how can we enhance beta power prior to these tasks? Rather than listening to music in the background during a cognitive task, which may or may not enhance performance, it might be worthwhile investigating whether a short period of *focused* listening to music can stimulate our brain, possibly via enhanced beta power, such that our performance in a subsequent cognitive task is enhanced. There is even some evidence that (focused) listening to music can enhance beta power (Nakamura et al., 1999), which could be monitored in experimental setups with EEG biofeedback methods (Egner and Gruzelier, 2004). However, more research is needed to establish the conditions under which music listening affects beta power, including the type of music, current mood of the listener and possibly social factors as well. In a similar vein, stable and transient inter-individual differences, contextual features and the type of assignment will continue to be important factors for predicting the performance in cognitive tasks. But maybe focused music listening has the power to get us “in the zone” in a way of which background music is incapable.

## Author contributions

The author confirms being the sole contributor of this work and approved it for publication.

### Conflict of interest statement

The author declares that the research was conducted in the absence of any commercial or financial relationships that could be construed as a potential conflict of interest.
